# Feasibility of Bamboo Sawdust as Sustainable Alternative Substrate for *Auricularia heimuer* Cultivation

**DOI:** 10.3390/jof11050387

**Published:** 2025-05-17

**Authors:** Ya-Hui Wang, Cong-Sheng Yan, Yong-Jin Deng, Zheng-Fu Zhu, Hua-An Sun, Hui-Ping Li, Hong-Yuan Zhao, Guo-Qing Li

**Affiliations:** 1 Key Laboratory of Horticultural Crop Germplasm Innovation and Utilization, Co-Construction by Ministry and Province, Institute of Horticultural, Anhui Academy of Agricultural Sciences, Hefei 230001, China; wangyahui@aaas.org.cn (Y.-H.W.); congshengyan@126.com (C.-S.Y.); 2 Institute of Vegetables, Anhui Academy of Agricultural Sciences, Hefei 230001, China; 3 Edible and Medicinal Mushroom Innovation Centre, Anhui Academy of Agricultural Sciences, Hefei 230001, China; 4 Dongzhi Black Fungus Industrial Technology Research and Development Centre, Chizhou 247200, China; 5 Sericulture Research Institute, Anhui Academy of Agricultural Sciences, Hefei 230001, China; iamdyjm@163.com; 6 Institute of Vegetable Crops, Jiangsu Academy of Agricultural Sciences, Nanjing 210014, China

**Keywords:** *Auricularia heimuer*, bamboo sawdust, mycelial growth, nutrient content, redox balance

## Abstract

With the increasing scarcity of traditional hardwood sawdust resources, developing sustainable substrates for edible fungi cultivation has become an urgent industrial priority. This study systematically evaluated the effects of bamboo sawdust substitutions (20%, 30%, 40%, and 50%) on mycelial growth, fruiting body development, and nutritional quality of *Auricularia heimuer*, while elucidating the underlying molecular mechanisms through transcriptome sequencing. The results demonstrated that bamboo substitution of ≤30% maintained normal mycelial growth and fruiting body differentiation, with 20% and 30% substitutions increasing yields by 5.30% and 3.70%, respectively, compared to the control. However, 50% substitution significantly reduced yield by 9.49%. Nutritional analysis revealed that 20–40% bamboo substitution significantly enhanced the contents of crude protein, polysaccharides, and essential minerals (calcium, iron, and selenium) in fruiting bodies. Transcriptome analysis identified upregulation of glycosyl hydrolase family genes and downregulation of redox-related genes with increasing bamboo proportions. Biochemical assays confirmed these findings, showing decreased oxidative substances and increased reductive compounds in mycelia grown with high bamboo content, which indicate disrupted cellular redox homeostasis. This study provides both a practical solution to alleviate the “edible mushrooms derived from lignicolous fungi–forest conflict” and fundamental insights into fungal adaptation mechanisms to non-wood substrates, thus establishing a theoretical foundation for the valorization of agricultural and forestry wastes.

## 1. Introduction

Edible mushroom production represents a major global agricultural industry and ranks fifth in total output after grains, cotton, fruits, and vegetables. This industry primarily utilizes agricultural, forestry, and livestock byproducts as nutrient-rich substrates containing valuable bioactive compounds [1,2]. Edible mushroom culture substrate refers to the material used for the nourishment and reproduction of mycelium, which, in turn, serves as the “soil” for the growth and reproduction of edible mushrooms [3,4].

The conventional cultivation substrate for wood-decomposing edible fungi is mainly hardwood sawdust, which provides optimal nutrient content and an appropriate carbon-to-nitrogen ratio [5]. Nevertheless, following more than a decade of accelerated expansion in the cultivation of black fungus (*Auricularia heimuer*) and other wood-decaying edible fungi, forest resources have become severely depleted [6]. This has led to an increasingly stark contradiction between industrial development and the scarcity of resources in what is known as the “mushroom–forest paradox” [7]. Consequently, the urgent need to develop alternative raw materials that can replace wood chips while maintaining the quality of mushrooms has emerged as the most pressing challenge in edible mushroom cultivation [8].

China possesses abundant bamboo resources with extensive bamboo forest distribution [9,10]. The country accounts for approximately 40% of the world’s bamboo (Bambusoideae) species, with bamboo timber production representing approximately one-third of the global total. The relatively short growth cycle of bamboo makes it an easily renewable resource conducive to the realization of sustainable use of bamboo, because developing bamboo shavings cultivation for edible fungi provides sufficient resource conditions [11,12]. Bamboo sawdust exhibits superior hemicellulose content and water retention capacity compared to conventional wood sawdust [13,14] while also providing essential amino acids, lipids, and minerals [10,15]. In the mountainous regions of southern Anhui Province, China, the bamboo material cannot be utilized in a meaningful way, and it is piled up directly at the felling operation area after felling, resulting in waste. Furthermore, bamboo demonstrates remarkable ecological competitiveness through its vigorous rhizome system, which inhibits the growth of other tree species [16,17]. From the perspectives of nutrient composition, resource utilization efficiency, and ecological impact, bamboo is an excellent substrate for the cultivation of edible mushrooms.

Currently, the development of edible mushrooms cultivated on bamboo-based substrates has demonstrated promising effectiveness in several aspects. Notably, while bamboo substrates can partially replace wood sawdust without compromising mushroom yield, they significantly enhance nutritional quality. However, considerable research still needs to be conducted before the technology can be applied to production at scale. Chutimanukul et al. [18] tested the utilization of rubber wood sawdust and bamboo sawdust, alone or as a substrate mixture, for the industrial-scale production of *Hericium erinaceus*., and the results showed that the *H. erinaceus* obtained by using 25% of rubber wood sawdust instead of rubber wood sawdust had the same yield and quality as substrates containing 100% rubber wood sawdust. Ye et al. [19] systematically assessed the feasibility of replacing wood sawdust with bamboo sawdust for cultivating *A. cornea* and its quality, and the results showed that it was feasible to replace the same proportion of wood sawdust in the control with 8% bamboo sawdust for *A. cornea* production. Partial replacement of wood sawdust with bamboo sawdust significantly (*p* < 0.05) increased the amino acid, ash, and protein contents and significantly decreased the crude fiber content of the mushroom substrate, and 8–16% of bamboo sawdust replacing wood sawdust also significantly increased the crude polysaccharide content of the substrate. Zeng et al. [20] used bamboo shavings to replace 50% of wood shavings in in *Lentinula edodes* cultivation (strains L808 and Zhexiang No. 6), and the results showed that the mycelial growth, total yield, and quality of shiitake mushrooms in the bamboo shavings culture medium were better than those in the pure wood shavings. Fu et al. [21] added 27% of moso bamboo processing waste to the *A. heimuer* culture substrate, and there was no significant difference in *A. heimuer* yield, substrate crude protein, and crude fat content compared with the control, which indicated that it was feasible to use bamboo waste instead of wood sawdust for *A. heimuer* culture. These successful cases collectively demonstrate bamboo sawdust’s potential as an alternative substrate for novel edible mushroom cultivation. However, it should be noted that successful implementations have only been achieved with relatively low bamboo sawdust substitution percentages.

The primary objective of this study was to investigate the physiological mechanisms responsible for yield reduction in edible mushrooms cultivated with high-proportion bamboo sawdust substrates. To achieve this, we aimed to perform the following: (1) characterize the transcriptomic profiles of *A. heimuer* mycelia under bamboo-rich cultivation conditions; (2) assess the impact on redox homeostasis through analysis of key oxidative stress markers; (3) evaluate associated changes in agronomic performance and quality parameters.

## 2. Materials and Methods

### 2.1. Fungal Strains

*A. heimuer* breed “Wanheimuer No. 1” (Registration No. 皖品鉴登字第2012003, Anhui Provincial Crop Variety Certification Committee), a high-quality variety cultivated from wild resources in southern Anhui for many years by systematic selection, belongs to medium–high temperature type varieties, it produces abundant clusters of thick, round to bowl-shaped fruiting bodies with uniform size, high gelatin content, excellent elasticity, and a deep brown color, as well as the yield as high as 0.50 kg/m^2^ [22]. The spawn is produced by the Edible Mushroom Team, Vegetables Research Institute, Anhui Academy of Agricultural Sciences, China, and the mushroom stick is produced by Huangshan Qingfeng Edible Mushroom Co., Huangshan District, Huangshan, China.

### 2.2. Media Formulation

The mass percentages of each component of the culture formulation for the spawn are as follows: wood sawdust 78%, wheat bran 20%, glucose 1%, gypsum 1%, and lime 1%. Mushroom-stick formulations were quantified with raw materials such as bran, gypsum, and lime and combined into four formulations with raw materials such as wood sawdust and bamboo (*Phyllostachys edulis*) sawdust as variables, and the conventional formulations were used as control (CK) during production in the experiment. All raw materials used in the formulations were fresh and free from mold contamination, with detailed compositions provided in Table 1.

### 2.3. Transcriptome Sequencing and Analysis

Total RNA was extracted from mycelium grown in different substrate formulations (with three biological replicates per treatment group) using TRIzol reagent (Invitrogen, Waltham, MA, USA). RNA quality was rigorously assessed by the following: (1) concentration and purity measurements using Nanodrop 2000 (Thermo Fisher Scientific, Waltham, MA, USA; acceptable ranges: 30–300 ng/μL, A260/280 = 1.8–2.2); (2) RNA Quality Number (RQN) determination using Agilent 5300 (Agilent, Santa Clara, CA, USA, required RQN > 6.5 for library construction). Polyadenylated mRNA was enriched using oligo (dT) beads, fragmented to ~300 bp, and reverse transcribed into cDNA for library construction with Illumina-compatible adapters. Sequencing was performed on DNBSEQ-T7 (MGI Tech Co., Ltd., Shenzhen, China) (100 bp paired-end) platforms following standard protocols. De novo assembly was conducted using Trinity (v2.13.2) with default parameters. The expression level of each gene is calculated using RNA-seq by Expectation Maximization (RSEM). The differentially expressed genes (DEGs) were selected out using the NOISeq method. According to the GO database (https://www.geneontology.org/, 17 January 2024) and KEGG pathway database (https://www.genome.jp/kegg/, 17 January 2024), the DEGs were classified by function and pathway. Then, the enrichment analysis of DEGs was performed by the phyper function. The transcriptome sequencing was performed by Shanghai Meiji Biomedical Technology Co., Ltd. (Shanghai, China), and the data analysis was based on its BioSignal Cloud Platform.

### 2.4. Oxidizing and Reducing Substances Determination

The levels of hydrogen peroxide (H_2_O_2_), oxygen free radical (O_2_^−^), nitric oxide (NO), reduced glutathione (GSH), and the activities of superoxide dismutase (SOD), peroxidase (POD), and catalase (CAT) enzymes in the mycelial cells of *A. heimuer* in each formulation were determined using the appropriate kit (Suzhou Grace Biotechnology Co., Ltd., Suzhou, China) and following the instructions outlined in the protocol.

Tissue sample preparation: Fresh mycelium (0.02 g, m) was weighed and homogenized in 2 mL (V) of pre-cooled extraction buffer using an ice bath. The homogenate was centrifuged at 12,000 rpm and 4 °C for 10 min. The resulting supernatant was collected and maintained on ice for subsequent analysis.

Determination of H_2_O_2_ content: First, 100 μL (V_1_) of the sample supernatant, 150 μL of Reagent 1, and 100 μL of Reagent 2 were sequentially added into an EP tube. The mixture was vortexed thoroughly and incubated at room temperature for 5 min. The absorbance (A) was then measured at 510 nm using a microplate reader, with the reaction system containing extraction buffer serving as the blank control. All treatments were performed in triplicate. The hydrogen peroxide content was calculated according to the following equation:

H_2_O_2_ content (μmol/g) = (A − 0.002) ÷ 6.97 ÷ (m × V_1_ ÷ V)

Determination of superoxide anion content: Reagent 2 and Reagent 3 were first mixed in equal proportions to prepare the reaction mixture. Then, 35 μL (V_1_) of sample supernatant and 35 μL of Reagent 1 were added to an EP tube and mixed thoroughly. After incubation at 37 °C for 10 min, the reaction mixture was added to the tube and vortexed. The reaction was allowed to proceed at 37 °C for an additional 5 min before the absorbance (A) was immediately measured at 540 nm using a microplate reader. The reaction system containing extraction buffer served as the blank control. All treatments were performed in triplicate. The O_2_^−^ content was calculated using the following equation:

O_2_^−^ content (μmol/g) = (A + 0.0011) ÷ 0.0482 ÷ (m × V_1_ ÷ V) × 2

Determination of Nitric Oxide (NO) Content: The sample supernatant was boiled for 5 min and then placed on ice. Subsequently, 20 μL of Reagent 1, 10 μL of Reagent 2, 5 μL of Reagent 3, and 60 μL of the boiled sample supernatant (V_1_) were sequentially added to an EP tube. The mixture was vortexed and incubated at 37 °C for 60 min. Then, 40 μL of Reagent 4 was added, followed by incubation at 37 °C for 30 min. Finally, 200 μL of a reaction mixture (prepared by mixing Reagent 5 and Reagent 6 in equal proportions) was added, and the reaction was allowed to proceed at 37 °C in the dark for 15 min. The absorbance (A) was measured at 530 nm. The absorbance (A_1_) of a system containing 0.1 μmol/mL NO standard (C) was also measured. The reaction system with distilled water served as the blank control. All treatments were performed in triplicate. The NO content was calculated according to the following equation:

NO content (μmol/g) = (C × V_1_) × A ÷ A_1_ ÷ (m × V_1_ ÷ V)

Determination of GSH Content: In an EP tube, 40 μL of sample supernatant (V_1_), 240 μL of Reagent 1, and 80 μL of Reagent 2 were sequentially added and immediately mixed. After standing for 5 min, the absorbance (A) was measured at 412 nm. The blank control consisted of 40 μL of sample supernatant and 320 μL of Reagent 1. The GSH content was calculated according to the following equation:

GSH content (μmol/g) = (A + 0.0035) ÷ 19.371 ÷ (m × V_1_ ÷ V)

Determination of SOD activity: Three EP tubes were prepared for the assay. Tube 1 received sequential additions of 140 μL of Reagent 1, 30 μL of Reagent 2, 30 μL of sample supernatant (V_1_), 15 μL of Reagent 3, and 160 μL of Reagent 4. Tube 2 contained 140 μL of Reagent 1, 30 μL of Reagent 2, 60 μL of distilled water, 15 μL of Reagent 3, and 160 μL of Reagent 4. Tube 3 was prepared with 140 μL of Reagent 1, 120 μL of distilled water, 15 μL of Reagent 3, and 160 μL of Reagent 4. After thorough mixing (total volume = V_2_), the reaction systems were incubated at room temperature in the dark for 30 min. The absorbance values were then measured at 450 nm and recorded as A_1_, A_2_, and A_3_ for tubes 1, 2, and 3, respectively. SOD activity was calculated using the following formula:

Formazan formation inhibition rate (R) = (A_2_ − A_3_) − A_1_] ÷ (A_2_ − A_3_)

SOD activity (U/g) = R ÷ (1 − R) × V_2_ ÷ (m × V_1_ ÷ V)

Determination of Peroxidase (POD) Activity: In an EP tube, 20 μL of sample supernatant (V_1_), 80 μL of Reagent 1, 280 μL of Reagent 2, and 20 μL of Reagent 3 were sequentially added and mixed. The absorbance (A_1_) was immediately measured at 470 nm, and the absorbance (A_2_) was measured again after 5 min (T). One unit of POD activity was defined as the amount of enzyme required to increase the absorbance by 0.5 per minute per gram of tissue in the reaction system. POD activity was calculated using the following formula:

POD activity (U/min/g) = (A_2_ − A_1_) ÷ (m × V_1_ ÷ V) ÷ 0.5 ÷ T

Determination of Catalase (CAT) Activity: In an EP tube, 10 μL of sample supernatant (V_1_), 70 μL of Reagent 1, and 20 μL of Reagent 2 were sequentially added and mixed. After incubation at room temperature for 5 min, 100 μL of Reagent 3 was added. Then, 10 μL of the mixture was transferred to a new EP tube and mixed with 900 μL of Reagent 1 and 290 μL of Reagent 4. After incubation at room temperature for 5 min, the absorbance (A) was measured at 510 nm. The blank control consisted of 10 μL of a mixture of Reagent 1 (20 μL), Reagent 2 (20 μL), and Reagent 3 (100 μL), mixed with 900 μL of Reagent 1 and 290 μL of Reagent 4, and incubated under the same conditions. The absorbance (A_1_) was measured at 510 nm after 5 min (T). CAT activity was calculated using the following formula:

CAT activity (μmol/min/g) = (A_1_ − A + 0.0025) ÷ 0.2093 ÷ (m × V_1_ ÷ V) ÷ T

### 2.5. Cultivation and Management

The test site was located in the edible mushroom demonstration base of Qingfeng Edible Mushroom Co. (N 31°50′47.75″; E 117°10′48.28″). The raw materials were then mixed in accordance with the formula, and the mixture was thoroughly stirred. The moisture content was to be 55%, while the pH level was within the range of 6.0–7.0. The bag size employed was a 17 cm × 55 cm polyethylene mushroom bag with a thickness of 0.055 cm purchased from Wuhan Jingnong Fungi Industry Co., Ltd. (Wuhan, China). Following the loading of the bag with 2 kg of material, it was securely tied at the mouth and placed in a sterilizing appliance at atmospheric pressure, with the temperature set to 100 °C. This process was continued for a duration exceeding 12 h in an FX-3 edible fungus sterilization chamber (Hubei Fuxing Machinery, Wuhan, China). Subsequent to this, the bag was transferred to a shaded area for cooling, resulting in a material temperature that did not exceed 28 °C. Thereafter, the material was inoculated. The inoculation was performed by creating unilateral perforation with three 2 cm diameter inoculation holes on one side. A 17 cm × 60 cm, 0.01 cm thick polyethylene plastic bag was then placed over the mushroom stick. Kept the culture room clean, well-ventilated, and dry. The configuration of the stacking should be modified in accordance with the temperature. At temperatures below 25 °C, the sticks should be arranged in three layers, with three sticks in the first layer positioned horizontally side by side, three sticks in the second layer arranged vertically, and three sticks in the third layer positioned horizontally, with appropriate gaps between the sticks in each layer. Conversely, at temperatures higher than 25 °C, the sticks should be arranged in a single layer in the incubation chamber. Control the temperature of the shed below 30 °C and remove the contaminated bags in time. Record the mycelial growth rate at the appropriate time, which is calculated as follows:

mycelial growth rate= (colony diameter on day 10 − colony diameter on day 5) ÷ 5

The time taken for complete colonization of mycelium in the sticks of each formula was meticulously recorded, and after a further seven days of incubation approximately 200 holes (0.5 cm in diameter and 1 cm in depth) were punched in each mushroom stick. Placed the punched mushroom sticks crosswise and diagonally on the wire, with a distance of about 10 cm between each stick, and twisted the sticks every 5 days. The field should be thoroughly watered before placing the sticks. The spraying process was initiated 2–3 days after the sticks were placed. Sprayed water a small number of times in the primary and young ear stage, 4–6 times a day, 5 min–10 min each time; the frequency and time of spraying water during the fruiting body growth stage shall be changed to 2–3 times a day, 30 min each time; stopped spraying water 1–2 days before harvesting. Fruiting body growth period, the highest temperature did not exceed 25 °C, could be in the daytime intermittent watering, watering amount to the ear piece completely unfolded when you can stop; temperature higher than 25 °C in the morning and evening spraying; high temperature shall not be sprayed. The fruit bodies were typically harvested at 70–90% developmental maturity, following the selective harvesting practice of collecting larger fruiting bodies while allowing smaller ones to continue growing. After harvesting and drying the ear pieces, we weighed the weight of the dried ear of each formula after 5 tides of harvesting and calculated the yield of a single mushroom stick.

### 2.6. Agronomic and Quality Trait Measurement

Agronomic traits included parameters such as mycelial growth rate, fruiting body size, fruiting body thickness, and yield. The size and thickness of fruiting bodies were determined using precision vernier calipers. Size was measured as the maximum diameter, and thickness was recorded at the central point of each fruiting body, with thirty replicates measured per treatment. Quality traits included crude protein, crude fat, crude polysaccharide, crude fiber, calcium, iron, zinc, and selenium [23,24,25,26].

The *A. heimuer* fruiting bodies thus harvested were subjected to drying in an oven at 40 °C, following which they were powdered using a crusher, and subsequently sieved (0.425 mm) to obtain the *A. heimuer* powder.

The crude protein content was determined by the Kjeldahl method using the fully automatic Kjeldahl nitrogen tester ATN-300 (Huazheng Analysis Instrument Technology Co., Ltd., Hangzhou, China), and the mass of crude protein was calibrated by the content of N in the samples. A quantity of 1.0000 g of *A. heimuer* powder was weighed and transferred into a dry 250 mL nitrogen flask. Copper sulfate (0.4 g), potassium sulfate (6 g), and sulfuric acid (20 mL) were then added, after which the flask was gently agitated. A small funnel was placed at the mouth of the flask, and the flask was positioned diagonally at an angle of 45 °C on an asbestos mesh with small holes. The heating was carried out with caution until the contents of all carbonization and foam had ceased. The fire was strengthened, and the liquid in the bottle was maintained at a slightly boiling temperature until the liquid turned blue-green and became clarified and transparent. The heating was continued for a period of 0.5 to 1 h. The nitrogen flask was then removed and placed on a cooling rack. Following this, 20 mL of water was added, and the substance was allowed to cool once more. Thereafter, the liquid was transferred to 100 mL of volumetric flasks. A small amount of water was then added to the nitrogen flasks, which were washed into the volumetric flasks. Volumed to 100 mL. Concurrently, the blank test was conducted. The nitrogen distillation device was installed, filled with water to two-thirds of the water vapour generator capacity, and a few glass beads, drops of methyl red ethanol solution, and a few millilitres of sulfuric acid were added, to keep the liquid orange-red during the distillation process, heated and boiled the water in the water vapour generator and kept it boiling. The next step was to add 10.0 ml of a 20 g/L boric acid solution and 1–2 drops of a mixed indicator (a 1 g/L solution of methyl red ethanol mixed with 0.5 g/L of bromocresol green ethanol solution in equal volume when ready for use) to the receiving bottle, insert the lower end of the condenser tube under the liquid surface, draw up 10 mL of the sample treatment solution, inject it into the reaction chamber, rinse a small amount of water out of the inlet port, then add 10.0 mL of sodium hydroxide solution (400 g/L), immediately tighten the glass stopper, and sealed the water to prevent air leakage. The screw clamp was securely fastened, and the distillation process was initiated. Following a distillation period of 10 min, the conical flask was positioned in such a manner that the terminal extremity of the condenser tube was positioned at a distance from the surface of the absorbing liquid. Thereafter, the distillation process was continued for a duration of one minute. Subsequently, the condenser tube extremity was rinsed with water in order to flush away any residuals, and then the distillation was stopped. Subsequent to this, the absorbent was titrated with a 0.1 mol/L hydrochloric acid standard titration solution until the colour turned grey-blue (volume of hydrochloric acid consumed of the sample is V_1_), and a reagent blank was prepared (volume of hydrochloric acid consumed of the blank is V_0_).

Crude protein content = (V_1_ − V_0_) × 0.1 × 0.014 × 6.25 × 100/1.0000/10

The crude fat content was extracted by Soxhlet extraction method, each 5 g of *A. heimuer* powder was weighted and wrapped in filter paper and then placed in a 250 mL weighted evaporation flask (m_1_), 100 mL of petroleum ether (30–60 °C) was then added, and a Soxhlet extracted was mounted to heat the extract for 8 h. The filter paper packet was taken out, and the sample was continued to be dried by rotary evaporation for a further 2 h in a rotary evaporation flask, and the residual petroleum ether was then evaporated in a fume hood overnight, and then dried in an oven at 60 °C to a constant weight of m_2_. This process was repeated on three separate occasions for each sample. The equation is as below:

Crude fat content (mg/g) = (m_2_ − m_1_)/5

The crude polysaccharide was extracted by hot water immersion; 1 g of *A. heimuer* sample powder was placed in a 500 mL conical flask, and 200 mL of distilled water was added to the flask and extracted at 100 °C for 2 h. After extraction, the sample was cooled to room temperature, centrifuged at 5000 rpm for 10 min in a 50 mL centrifuge tube, and the supernatant was collected in an evaporation dish and evaporated to 30 mL in a water bath at 100 °C, and then up to 40 mL of the solution was prepared in a volumetric flask. Moreover, 2 mL of the above solution was accurately measured and then put into a 50 mL centrifuge tube, 8 mL of anhydrous ethanol was added and centrifuged at 5000 rpm for 10 min, and then the precipitate was washed with anhydrous ethanol for 1 h. The precipitate was washed with anhydrous ethanol once, placed in an oven at 70 °C for 1 h, and the precipitate was dissolved with 10 mL of distilled water, and this was repeated three times for each sample, and the polysaccharide content was detected by the concentrated sulfuric acid-anthracenone method [27]. The polysaccharide contents of the *A. heimuer* samples were calculated by regression equations based on the absorbance values of the detected samples.

The crude fiber content was determined using a cellulose tester F800, weighing 1 g of powdered *A. heimuer* fruiting body samples to be tested, and then into a crucible in which diatomaceous earth was added, and after 1.25% sulfuric acid and 1.25% KOH decoction on the cellulose tester, it was placed in an oven at 130 °C until constant weight and weighed as m_1_, and then placed in a muffle furnace for incineration at 500 °C for 4 h, and then cooled down to room temperature and weighed as m_2_.

Crude fiber content (mg/g) = (m_1_ − m_2_)/1

### 2.7. Statistical Analysis

The data were subjected to analysis of variance (ANOVA) using the Excel and DPS software, version 15.10. ANOVA and Fisher’s protected least significant difference (LSD) test were used to determine the significance (*p* ≤ 0.05) of differences between treatment means.

## 3. Results

### 3.1. Mycelial Growth and Transcriptome Analysis

**Figure 1:**
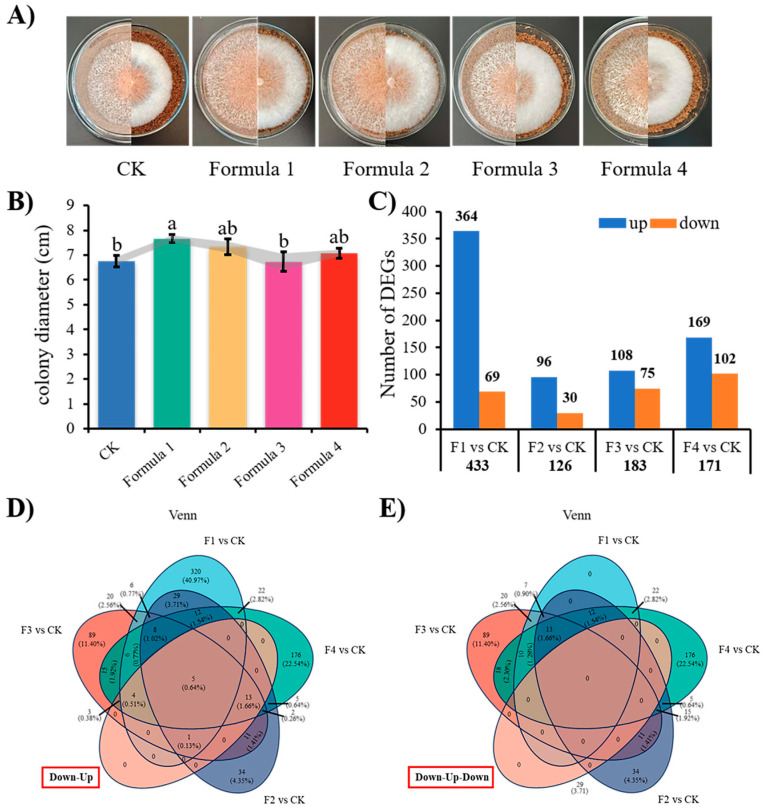
Growth and transcriptome gene expression analysis of *A. heimuer* “Wanheimuer No. 1” in various formulations: (**A**) growth of the “Wanheimuer No. 1” in the formulas of bamboo sawdust; (**B**) colony diameter of “Wanheimuer No. 1” in different bamboo sawdust formulations; (**C**) number of DEGs of “Wanheimuer No. 1” in different formulations; (**D**) overlapping relationships between gene sets of down–up and multiple formulations of differential genes; (**E**) overlapping relationships between gene sets of down–up–down and multiple formulations of differential genes. Different letters above the chart columns indicate significant differences among treatments (*p* ≤ 0.05).

The experiment evaluated four bamboo sawdust substitution formulations alongside a conventional wood sawdust control for cultivating “Wanheimuer No. 1”. Growth rate analysis revealed a characteristic unimodal response, with mycelial growth initially increasing before declining as the bamboo sawdust percentage increased (Figure 1A,B). Transcriptome sequencing of mycelia grown in five formulations showed that Formula 1 had more differentially expressed genes, with upregulated genes consistently outnumbering downregulated genes in mycelium grown in each bamboo sawdust formulation (Figure 1C). Through systematic trend analysis, we identified two distinct gene expression patterns: a 26-gene “down–up” set correlating with bamboo percentage (Figure 1D) and a 349-gene “down–up–down” set mirroring the mycelial growth rate trajectory (Figure 1E). Validation by qPCR analysis of five representative DEGs (four upregulated and one downregulated; Supplementary Table S1) confirmed strong concordance between transcriptomic and qPCR results (Supplementary Figure S1), supporting the reliability of our sequencing data.

### 3.2. Down–Up–Down Gene Set Analysis

**Figure 2:**
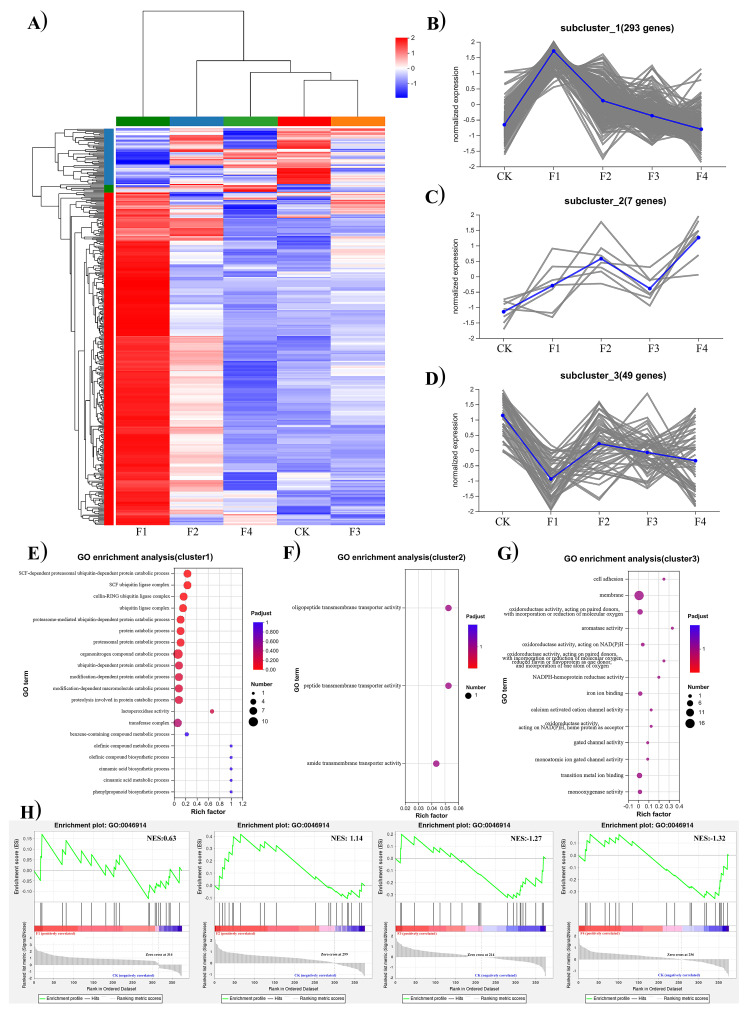
Clustering and functional enrichment analysis of down–up–down gene sets with a tendency of gene expression to be initially up- or downregulated and then recovered. (**A**) Heatmap visualization of expression patterns in down–up–down gene sets; (**B**) Expression pattern of subcluster 1 genes; (**C**) Expression pattern of subcluster 2 genes; (**D**) Expression pattern of subcluster 3 genes; (**E**) Bubble plot of GO enrichment analysis for subcluster 1; (**F**) Bubble plot of GO enrichment analysis for subcluster 2; (**G**) Bubble plot of GO enrichment analysis for subcluster 3; (**H**) Gene set enrichment analysis (GSEA) reveals functional enrichment of the down–up–down gene sets.

The down–up–down gene set referred to the tendency of the gene expression to be initially up- or downregulated, and it recovered as the percentage of bamboo sawdust in the substrate increased. The 349 differentially expressed genes were classified into three clusters according to their expression patterns (Figure 2A). Subcluster 1 contained the highest number of differentially expressed genes (DEGs) (n = 293), displaying a distinct expression pattern marked by significant upregulation under the 20% bamboo sawdust treatment, while no significant differential expression was observed relative to the control at higher bamboo sawdust percentage (Figure 2B). Gene Ontology (GO) enrichment analysis demonstrated that these DEGs were primarily enriched in metabolic and biosynthetic pathways (Figure 2E). Subcluster 2 displayed the lowest number of DEGs (n = 7), showing consistent upregulation under both 20% and 30% bamboo sawdust treatments. Notably, this upregulation pattern disappeared at 40%, where gene expression levels became statistically indistinguishable from the control (Figure 2C). Functional annotation through GO enrichment analysis identified transmembrane transport as the most significantly enriched biological process among these DEGs (Figure 2F). Subcluster 3 contained 49 DEGs that were significantly downregulated in the 20% bamboo sawdust treatment but showed no differential expression relative to controls at higher percentages (Figure 2D). GO enrichment analysis revealed these genes were predominantly enriched in cell membrane-related functions (Figure 2G). Gene set enrichment analysis (GSEA) revealed that the down–up–down gene sets in the mycelia of “Wanheimuer No. 1” cultured with bamboo sawdust demonstrated significant enrichment for genes involved in transition metal ion-binding functions (GO: 0046914) compared to conventional formulation. Notably, the enrichment significance of transition metal ion-binding gene sets exhibited a strong positive correlation with increasing bamboo sawdust percentage (Figure 2H).

### 3.3. Down–Up Gene Set Analysis

**Figure 3:**
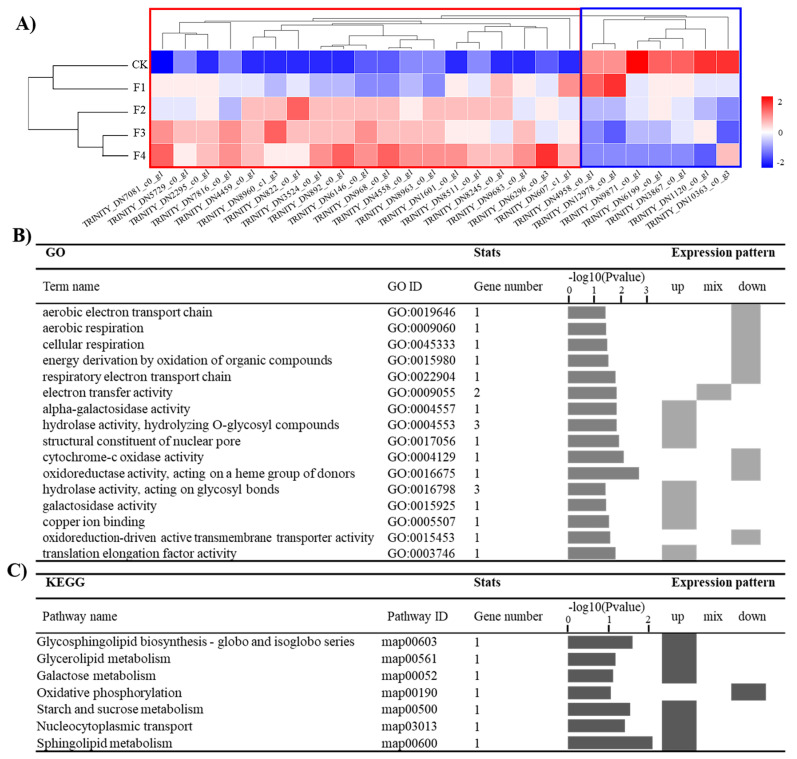
Clustering and functional enrichment analysis of down–up gene sets with a trend of gradual upregulation or gradual downregulation of gene expression. (**A**) Heatmap visualization of expression patterns in down–up gene sets, Gene expression patterns: Red boxes—upregulation with higher bamboo sawdust; Blue boxes—downregulation with increasing sawdust bamboo percentage. (**B**) GO enrichment analysis for down–up gene sets. (**C**) KEGG enrichment analysis for down–up gene sets.

The gene set designated as “down–up” genes showed a trend of gradual upregulation or gradual downregulation of gene expression with the increase of bamboo sawdust percentage in the substrate, which contained nineteen significantly upregulated and seven significantly downregulated expressed genes (Figure 3A). Functional annotation through GO and KEGG enrichment analyses revealed that these 26 genes were primarily associated with two key biological processes in *A. heimuer* “Wanheimuer No. 1”, and the genes related to hydrolase activity were upregulated genes, and the genes related to oxidation-reduction reaction were downregulated genes (Figure 3B,C).

### 3.4. Mycelial Redox State Analysis

**Figure 4:**
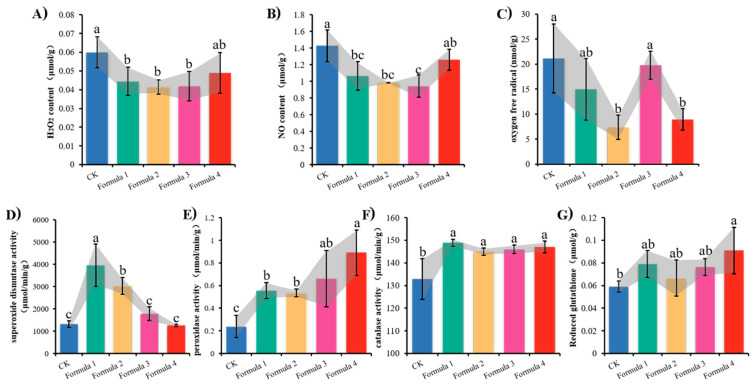
Oxidizing and reducing substances content assay of *A. heimuer* “Wanheimuer No. 1” in different formulations: (**A**) hydrogen peroxide (H_2_O_2_) content in *A. heimuer* mycelium; (**B**) nitric oxide (NO) content in *A. heimuer* mycelium; (**C**) oxygen free radical (O_2_^−^) content in *A. heimuer* mycelium; (**D**) superoxide dismutase (SOD) enzyme activity in *A. heimuer* mycelium; (**E**) peroxidase (POD) enzyme activity in *A. heimuer* mycelium; (**F**) catalase (CAT) enzyme activity in *A. heimuer* mycelium; (**G**) reduced glutathione (GSH) content in *A. heimuer* mycelium. Different letters above the chart columns indicate significant differences among treatments (*p* ≤ 0.05).

The activity of oxidizing and reducing substances in the cell, as well as the ratio between them, is pivotal in determining the redox state of the cell. It has been established that the overexpression of oxidizing or reducing substances within the cell can disrupt the redox balance. Our quantitative analysis of oxidative stress markers and antioxidant systems revealed distinct patterns: In bamboo sawdust formulations, mycelial cells showed significantly decreased levels of hydrogen peroxide (H_2_O_2_), superoxide anion (O_2_^−^), and nitric oxide (NO) compared to the wood sawdust control (Figure 4A–C). Conversely, these cells exhibited enhanced activity of key antioxidant enzymes—superoxide dismutase (SOD), peroxidase (POD), and catalase (CAT)—along with elevated reduced glutathione (GSH) content (Figure 4D–G). The results demonstrated that the bamboo sawdust formulation reduced the content of oxidizing substances and increased the content of reducing substances in the mycelial cells of *A. heimuer* compared to the wood sawdust formulation, suggesting that the cultivation of *A. heimuer* with bamboo sawdust may have affected the redox balance in the mycelial cells.

### 3.5. Bamboo Sawdust Cultivated Wood Ear

**Figure 5:**
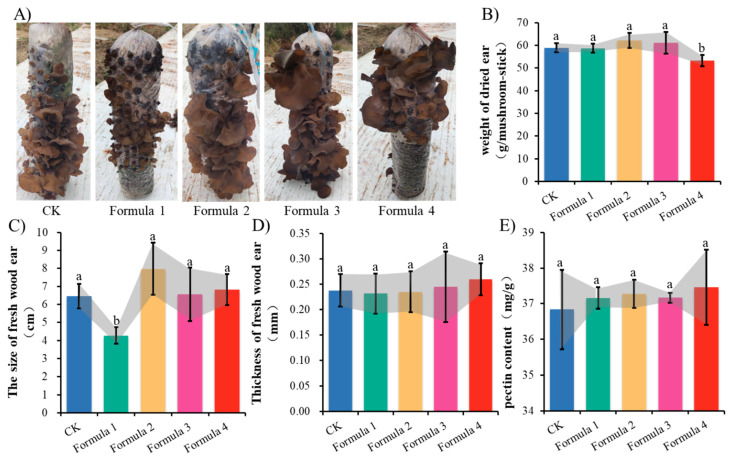
Agronomic traits of *A. heimuer* “Wanheimuer No. 1”: (**A**) phenotypes of “Wanheimuer No. 1” in different formulations; (**B**) weight of dried fruiting bodies in different formulations; (**C**) the size of fresh fruiting bodies; (**D**) thickness of fresh fruiting bodies; (**E**) pectin content of dried fruiting bodies. Different letters above the chart columns indicate significant differences among treatments (*p* ≤ 0.05).

Analysis of agronomic traits demonstrated significant impacts of bamboo sawdust incorporation on “Wanheimuer No. 1” cultivation. The substrate formulations differentially affected primordial differentiation (Figure 5A) and yield characteristics (Figure 5B), with primordia quantity following a distinct biphasic response-initial stimulation followed by inhibition-as bamboo percentage increased. As the percentage of bamboo sawdust increased, the number of primordia exhibited a trend of initial increase followed by a decrease, with Formula 1 demonstrating the latest primordia formation timing. In contrast, critical quality parameters of fruiting bodies, including size (Figure 5C), thickness (Figure 5D), and pectin content (Figure 5E), remained largely unaffected across all experimental formulations.

### 3.6. Nutritional Analysis of Wood Ear

**Figure 6:**
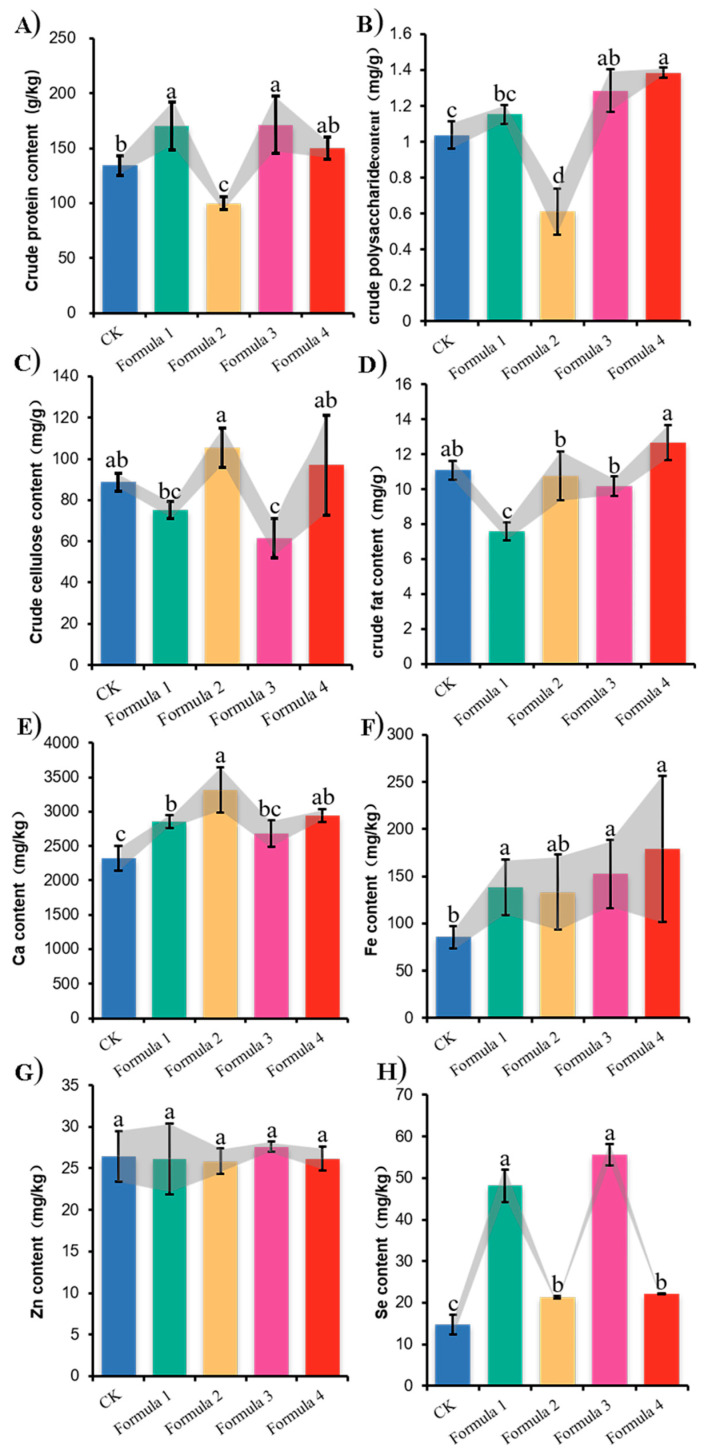
Nutrient content assay of *A. heimuer* “Wanheimuer No. 1” in different formulations: (**A**) crude protein content; (**B**) crude polysaccharide content; (**C**) crude fiber content; (**D**) crude fat content; (**E**) calcium content; (**F**) iron content; (**G**) zinc content; (**H**) selenium contents. Different letters above the chart columns indicate significant differences among treatments (*p* ≤ 0.05).

In the sulfuric acid-anthraquinone method, the standard curve was plotted with different concentrations of glucose, and the aldehydes formed by dehydration of glucose in the presence of sulfuric acid condensed with anthraquinone to form derivatives with a maximum absorption peak at 620 nm. Taking the absorbance value as the horizontal coordinate and the mass (mg) as the vertical coordinate, the regression equations and correlation coefficients were obtained as follows: y = 0.0513x − 0.0126, R^2^ = 0.9939. The determination of nutrient composition (Figure 6) showed that bamboo sawdust supplementation significantly enhanced several nutritional components of *A. heimuer* fruiting bodies in a formulation-dependent manner. While all bamboo-containing formulations showed improved crude protein, crude polysaccharide, calcium, iron and selenium contents compared to the control, Formulation 3 exhibited the most pronounced effects, significantly increasing crude protein (Figure 6A), crude polysaccharides (Figure 6B) and selenium (Figure 6H) while simultaneously reducing crude fiber content (Figure 6C). Formulation 1 also showed significant improvements in crude protein (Figure 6A), selenium (Figure 6H), and iron (Figure 6F), though it yielded lower crude fat content (Figure 6D). Mineral analysis revealed that calcium levels were significantly elevated in Formulations 1, 2, and 4 (Figure 6E), while iron content increased notably in Formulations 1, 3 and 4 (Figure 6F). Interestingly, zinc content remained statistically unchanged across all formulations (Figure 6G).

## 4. Discussion

Traditional mushroom cultivation relies heavily on broadleaf trees as growth substrates, leading to over-deforestation, the reduction of natural forests, and damage to biodiversity. The core of this conflict lies between the economic demands of mushroom production and the ecological goals of forest conservation. To address this issue, researchers have recently explored using agricultural and forestry waste as alternative substrates. This study investigated the feasibility of cultivating *A. heimuer* using bamboo sawdust instead of wood chips, aiming to reduce dependence on natural forests and promote sustainable industry development. The purpose of this experiment was to test the growth and development of *A. heimuer*, defined as a broadleaf wood-decaying fungus, in bamboo sawdust to provide data for the rational use and application of bamboo sawdust, a new culture substrate for *A. heimuer*. In the four substrate formulations we established, the mycelium of the wood ear could grow normally, the mycelium was white and vigorous, and the fruiting bodies could be differentiated and developed normally; at the same time, existing studies also proved that bamboo sawdust can be used to successfully cultivate edible mushrooms [28], which indicates that bamboo sawdust can be used as a new type of substrate for cultivating wood ear. The wood ear cultivated with bamboo sawdust had different degrees of improvement in yield and nutrient content, although some indexes were less significant compared with the control. The yields of *A. heimuer* cultivated with 20% and 30% bamboo sawdust substitution were 5.30% and 3.70% higher than the control, respectively, though not statistically significant. However, the 50% substitution group showed a significant 9.49% yield reduction compared to the control (*p* < 0.05), indicating that higher bamboo sawdust proportions negatively affect yield, which aligns with previous studies [19]. As observed in Figure 5A, the number of auricle differentiation decreased with increasing bamboo sawdust content, potentially explaining the yield reduction. The crude protein, polysaccharide, and selenium content exhibited similar trends across formulations. These nutritional components were significantly higher in the 20% and 40% substitution groups compared to the control (*p* < 0.05). However, the 30% group showed a significant decrease in these components relative to both the 20% and 40% groups, with crude protein and polysaccharide content even falling below control levels. This phenomenon may be attributed to accelerated fruiting body development in Formula 2, where basidiospore release potentially carried away these nutrients during maturation. Previous studies confirm that selenium in edible fungi primarily exists as selenoproteins and selenopolysaccharides, explaining its correlation with protein and polysaccharide content [29].

Transcriptomic analysis of *A. heimuer* mycelium cultivated in bamboo sawdust formulations identified two distinct gene expression patterns. The down–up gene set exhibited expression profiles correlating with bamboo sawdust concentration, containing 19 upregulated genes, with six functionally annotated. These included one gene annotated as NAD (P)-binding protein (TRINITY_DN6296_c0_ g3), one gene was annotated as MFS multidrug transporter (TRINITY_DN2295_c0_g1), one gene was annotated as WD40 repeat-like protein (TRINITY_DN607_c1_g1), and three genes were annotated as glycoside hydrolase family (GH) genes (TRINITY_DN8511_c0_g1, TRINITY_DN5729_c0_g1, TRINITY_DN968_c0_g1). TRINITY_DN6296_c0_g3 is annotated as the gene encoding the NADPH-dependent enoyl-acyl carrier protein, which has been studied in bacteria and catalyzes the last step of the bacterial type II fatty acid synthesis (FASII) cycle to reduce the enoyl-ACP to fully saturated acyl-ACP, which has been implicated in the bacterial response to the broad-spectrum antimicrobial agent triclosan [5-chloro-2-(2,4-dichlorophenoxy)phenol] (TCL) [30,31]. However, it has not been reported in fungi because its expression pattern was consistent with the addition of bamboo sawdust (R^2^ = 0.6923). It was hypothesized that it might be related to the adversed growth of *A. heimuer*, and its correlation with the crude fat content of *A. heimuer* was low (R^2^ = 0.3021). It needs to be further verified whether the gene is related to the adversed growth or the accumulation of fatty acids. The other gene, TRINITY_DN2295_c0_g1, is annotated as an MFS multidrug transporter; MFS proteins selectively transport a broad spectrum of substrates across membranes and play a critical role in multiple physiological processes [32,33]. However, in some cases, MFS transporters are also believed to act as drug transporters using a proton gradient, consequently conferring multidrug resistance (MDR) [34]. MFS transporters have been shown to take part in multidrug resistance in fungi since they are able to act as a drug H+ antiporter in microorganisms [32]. Current evidence indicates that the MFS transporter can also indirectly regulate internal pH and stress response mechanisms in fungi [35]. WD40 repeat proteins play an important role in diverse cellular functions [36]. Three genes were annotated as GH family genes, and the addition of bamboo sawdust increased the cellulose content of the substrate, so it is most likely that these three genes are key enzymes for cellulose degradation in *A. heimuer*, of which TRINITY_DN5729_c0_g1 was significantly upregulated and expressed in the high percentage of bamboo sawdust (40% and 50%), and was annotated as a GH5 protein, which was previously called cellulase family A [37]. Research has demonstrated the presence of additional cellulose-like substances in the residual chaff following the harvesting of *A. heimuer*. The efficient utilization of substrate is one of the key factors in improving mushroom yield. One of the essential questions affecting the utilization of substrates is how lignocellulose is degraded. Therefore, in-depth studies on the composition and mechanism of CAZyme can lay the groundwork for breeding edible mushrooms that are efficient in the degradation of lignocellulose [38]. Thus, three genes of *A. heimuer* cultivated on bamboo sawdust, which have been upregulated, can be developed for the selection of high-quality and high-yielding strains of *A. heimuer*.

There are also seven genes in the down–up gene set that were significantly downregulated with the increasing percentage of bamboo sawdust, and the GO and KEGG annotation analysis of these genes showed that they are related to oxidation-reduction reactions, with all of these genes being downregulated in expression (Supplementary Figure S2). The activity of oxidation-reduction reactions and their dynamic balance is one of the most fundamental and important biochemical reactions in living organisms. The activity of intracellular oxidizing substances—such as hydrogen peroxide, superoxide ions, nitric oxide, and reducing substances, which include glutathione reductase, NADPH, and vincristine—and the ratio between them determine the oxidation-reduction state of cells [39,40,41]. During cell stimulation or lesion, the expression of oxidizing substances or reducing substances in excess will disrupt the oxidation-reduction equilibrium of the cell and cause the oxidation-reduction stress, which will lead to the physiological and pathological changes in the cell and the organism [42,43,44]. In the present study, the majority of the genes comprising the oxidative phosphorylation pathway were found to be significantly downregulated in “Wanheimuer No. 1” grown with bamboo sawdust as the proportion of bamboo sawdust increased. Furthermore, the content of oxidized substances decreased, the content of reduced substances increased in the mycelial cells of *A. heimuer* cultured with bamboo sawdust, and the ratio of oxidized substances to reduced substances changed compared with that of the wood sawdust formulation. Consequently, it can be hypothesized that the elevated content of bamboo sawdust disrupts the redox balance in the mycelium of *A. heimuer*, thereby affecting its growth and development.

The GSEA was performed on the set of down–up–down genes from each treatment, and all were enriched in the transition metal ion binding unit. Transition metals interact with a large proportion of the proteome in all forms of life, and they play mandatory and irreplaceable roles [45,46]. This unit has been studied in maize and shown to be associated with the defense response in maize [47]. We then postulated that the differential expression of the transition metal ion binding unit of the mycelium of *A. heimuer* grown in a high bamboo sawdust percentage is also the result of the adverse response of *A. heimuer* to environmental stress. Therefore, the high percentage of bamboo sawdust is unsuitable for the cultivation of the *A. heimuer* strain, which corresponds to the disruption of the balance of the *A. heimuer* mycelial redox reaction. The high percentage of bamboo sawdust represents an adversity for *A. heimuer* growth. This may be due to the high fiber content, which influences the carbon-to-nitrogen ratio of the formulation. Alternatively, it may be due to the high-water retention of bamboo sawdust, which makes the substrate poorly permeable. Further investigation is required to determine the underlying cause.

**Figure 7:**
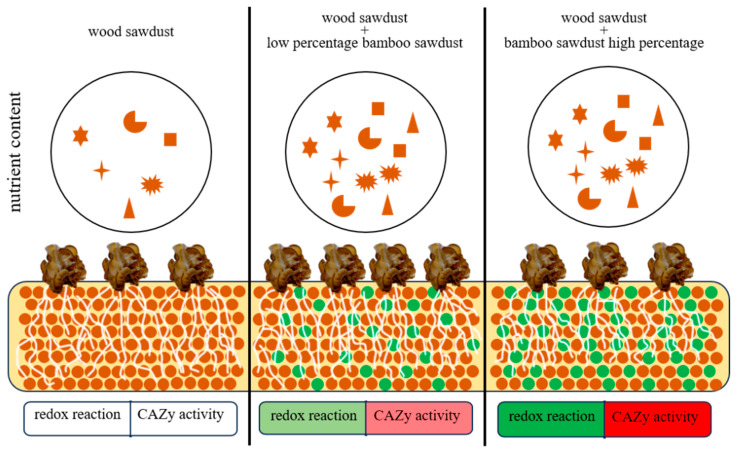
Modeling the growth and development of *Auricularia heimuer* in a bamboo sawdust formulation. As the percentage of bamboo sawdust increased the nutrient content of *A. heimuer* increased (brown dots represent wood sawdust, green dots represent bamboo sawdust), the genes related to the activity of carbohydrate-active enzymes were upregulated and expressed (red, color shades represent intensity), and the genes related to the redox reaction were downregulated and expressed (green, color shades represent intensity).

The final conclusions demonstrate that the redox balance status of *A. heimuer* mycelia is significantly affected as the proportion of bamboo sawdust increases. Although high bamboo sawdust content is unsuitable for conventional *A. heimuer* cultivation, moderate supplementation (20–30%) can improve both yield and quality characteristics of fruiting bodies. This study confirms the feasibility of bamboo sawdust cultivation for *A. heimuer*, but the addition ratio must be strictly controlled; when bamboo sawdust exceeds 50%, a significant redox imbalance occurs in the mycelial system (Figure 7). In contrast, an appropriate proportion (20–30%) not only reduces the use of hardwood sawdust and enhances bamboo utilization efficiency but also promotes the accumulation of bioactive components such as crude protein in fruiting bodies. It should be noted that the current research data remain preliminary. Future studies will focus on the following directions: (1) Mechanistic exploration: Elucidating how bamboo sawdust enhances the biosynthesis of key nutritional components (particularly polysaccharides and proteins) in *A. heimuer*; (2) Redox imbalance mechanisms: Systematically investigating the molecular mechanisms underlying mycelial redox dysregulation induced by high bamboo sawdust ratios, with emphasis on dynamic changes in key reactive oxygen species (ROS)-metabolizing enzymes; (3) Strain screening and genetic analysis: Targeted screening of superior strains with redox stress tolerance and functional characterization of cellulose-degrading genes (e.g., GH5 family); (4) Sustainable cultivation models: Developing optimized composite substrates combining bamboo sawdust with other agroforestry wastes (e.g., fruit tree branches, bagasse) to advance sustainable cultivation of wood-decaying edible fungi.

## Figures and Tables

**Table 1:** Cultivation media formula for *Auricularia heimuer* and content of N, C, and C/N of cultivation substrates.

Formula	Wood Sawdust (%)	Bamboo Sawdust (%)	Wheat Bran (%)	Gypsum (%)	Travertine (%)	C (%)	N (%)	C/N
CK	86	0	12	1	1	26.36	0.756	34.86
1	66	20	12	1	1	26.24	0.773	33.96
2	56	30	12	1	1	26.18	0.781	33.52
3	46	40	12	1	1	26.12	0.789	33.09
4	36	50	12	1	1	26.07	0.798	32.68

## Data Availability

The data underlying this article are available in the article and in its online Supplementary Materials. The RNA-seq repository names and accession numbers are as follows: https://www.ncbi.nlm.nih.gov/ (accessed on 17 January 2024) and PRJNA1120646.

## Supplementary Materials

The following supporting information can be downloaded at https://www.mdpi.com/article/10.3390/jof11050387/s1, Figure S1: Validation of transcriptome data using qPCR; Figure S2: KEGG enrichment analysis of the oxidative phosphorylation pathway of differentially expressed genes; Table S1: Primer sequences in this study. Reference [48] is cited in the supplementary materials.
