# Supplementary material for: Feasibility of Bamboo Sawdust as Sustainable Alternative Substrate for Auricularia heimuer Cultivation
Source: J Fungi (Basel). 2025 May 17;11(5):387. doi: 10.3390/jof11050387 (PMC12113283; doi:10.3390/jof11050387)
Supplement: Supplementary file 1 [file jof-11-00387-s001.zip › jof-3540424-supplementary.pdf]

Table S1. Primer sequences in this study

Gene name	Primer name	sequences	length
18S Reference Genes(Y, FJ, WJ, M, & CS, 2020)	18SrRNAF	CTGGCTCTGTCAGTGTAG	164
	18SrRNAR	TCCGATAACGAACGAGAC	
TRINITY-DN1601-c0-g1	1610F	TCACGGTCGTCCAGCAGT	290
	1610R	GATCGAGCCCAGCACAGA	
TRINITY-DN892-c0-g1	892F	CGGTTGCTACAGACAATACGG	239
	892R	GGACAACGCCAAGCAGAAT	
TRINITY-DN8963-c0-g1	8963F	GGCTTCGCAACATCACGG	117
	8963R	ATCGCATTTCATTTCTCCTTCA	
TRINITY-DN968-c0-g1	968F	CGCCATCGAGAACTACCG	189
	968R	CGGAGACGGACTCTGGGTT	
TRINITY-DN4958-c0-g1	4958F	GTGTATCCTGGCATCTATTTC	221
	4958R	ATGGCGGTCTTATTCTGG	

Zhang Y, Z., FJ, Y., WJ, S., M, F., & CS, W. (2020). Screening of reference genes for qRT-PCR amplification in *Auricularia heimuer*. *Mycosystema, 39*(8), 1510-1519.

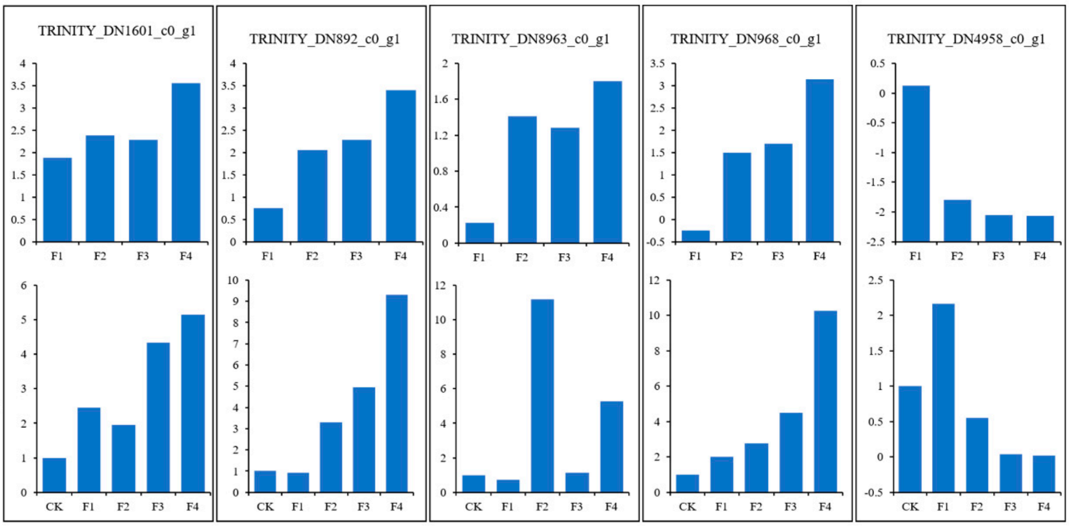

Figure S1: Validation of transcriptome data using qPCR

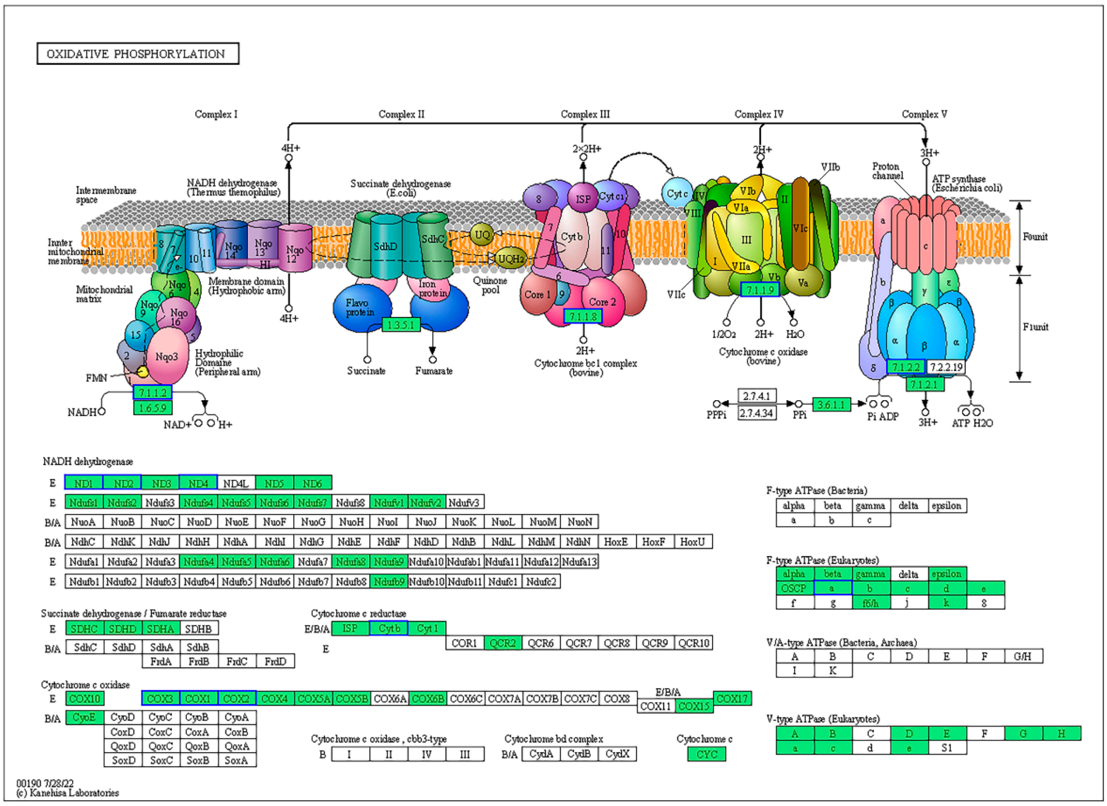

Figure S2: KEGG enrichment analysis of the oxidative phosphorylation pathway of differentially expressed genes
